# Afternoon Napping Durations in Chinese Population Over 60 Years Old: Longitudinal Associations With Cognitive Performance

**DOI:** 10.3389/fpubh.2022.911498

**Published:** 2022-07-07

**Authors:** Chao Li, Yan Yan

**Affiliations:** Department of Epidemiology and Medical Statistics, Xiangya School of Public Health, Central South University, Changsha, China

**Keywords:** cognitive decline, afternoon napping, longitudinal association, old adults, risk

## Abstract

**Introduction:**

Previous studies indicated inconsistent associations between daytime napping and cognitive decline. This study aimed to examine the associations between self-reported changes in napping and longitudinal cognitive performance.

**Methods:**

A national representative sample of 4,024 participants over 60 was obtained from the China Health and Retirement Longitudinal Study from 2011 to 2018. Afternoon napping and potential factors were collected by a questionnaire. Cognitive performance was assessed on three aspects. The generalized additive models and generalized estimating equations were used to examine relationships between daytime napping and longitudinal global cognition, and generalized linear models were used to examine the longitudinal associations between change in napping at four waves and cognition in wave 4.

**Results:**

After controlling the potential confounders, participants with afternoon napping were significantly related to better global cognition than no nappers at baseline. A change from short napping to no/long napping was associated with worse cognitive performance (β = −2.593, *P* < 0.001). A move from no napping to short/long napping was also associated with lower cognition scores (β = −0.694, *P* < 0.001). For participants with moderate napping, a >30 min increase (β = −1.558, *P* < 0.001) in afternoon napping was associated with worse cognitive function in wave 4.

**Conclusion:**

We observed that adults over 60 years old with napping <30 min per day may be at lower risk of cognitive decline. Change in napping, especially a move to extreme napping would be a risk marker underlying health conditions that impact cognition or go along with cognitive decline.

## Introduction

All around the world, dementia places a large burden on both caregivers and adult patients aged over 60 years old (1). The World Health Organization suggested that the number of people living with dementia was estimated to increase to 115.4 million by 2050 (2). 13.26 percent of the total Chinese adults were over 60 years old in 2010, and it is projected to grow to 400 million by 2050 (3). Given that aging is associated with negative health outcomes including cognitive impairment and dementia (4), which would cause an increase in the population burden of disability and mortality in old people and a heavy psychosocial and economic burden for families and society (5). China bears a heavy burden of Alzheimer's disease costs, which could greatly influence the estimates of Alzheimer's disease costs worldwide (6). Additionally, there is no effective treatment for dementia, it is essential for preventive strategies (7). Since dementia has a long preclinical phase in which accelerated cognitive decline could be regarded as a key marker (8). It is of great significance for identifying the modifiable risk factors which could contribute to preventing cognitive decline. The current research focused on the prevention of cognitive decline through risk factor identification and modification, with a growing interest in sleep (9, 10).

With the advancement of age, sleep patterns and circadian rhythm change (11). The prevalence of habitual daytime napping among old adults is significantly higher than in younger individuals (12). Furthermore, afternoon napping is defined as a part of healthy life in Chinese old adults from a cultural perspective. Few studies (13–16) focused on the association between napping and cognitive decline, especially for older people, but the relationship between afternoon napping and cognition was inconsistent (17, 18). Cai et al. (19) presented that afternoon napping was related to better cognitive performance, and Lin et al. (14) indicated that short afternoon naps (<30 min per day) could be associated with better cognitive performance in old people, but Owusu et al. (13) found that afternoon napping related to poorer cognitive performance in elders. Meanwhile, only one study (15) showed that keeping the duration of napping <90 min per day was associated with better cognitive performance after 2 years of follow-up. Lack of study uses data from long follow-ups to test the effects of baseline afternoon napping duration and the changes in afternoon napping on later cognitive performance among old adults.

To extend previous work, we examined the associations of baseline afternoon napping and changes in napping duration from baseline to wave 4 with changes in cognitive function after 8 years by using the China Health and Retirement Longitudinal Study (CHARLS) datasets. According to the previous research conducted on the impacts of napping, we divided afternoon napping into four groups: no napping, short napping, moderate napping, and long napping. We hypothesized that nappers at baseline would have better cognitive performance than non-nappers over the period, moderate nappers at baseline would maintain better cognitive function over the follow-ups, and groups that became or remained non-napping, short napping, or long napping would have worse cognitive performance compared to people in moderate napping groups across the four waves. To verify the hypothesis, we used a large-scale national cohort CHARLS which was representative of Chinese over 45 years old.

## Methods

China Health and Retirement Longitudinal Study is a national cohort study that is representative of Chinese residents aged 45 and older. This study used data from Chinese adults over 60 years old from CHARLS during 2011–2018. Participants from CHARLS were randomly selected using a multistage probability sampling design and a proportional sampling method (20). CHARLS was conducted and followed up every 2 years on the Chinese residents and their families (21) through self-report questionnaires and interviews.

This present study used data from waves 1 through wave 4, conducted in 2011, 2013, 2015, and 2018. A total of 17,708 individuals were included and the response rate was 80.5% at baseline. A total of 15,788 individuals with follow-up data were collected in the second survey, 15,333 individuals collected in the third wave, and 19,817 in wave 4. The sample in this study was restricted to respondents over 60 years old at baseline, provided self-reported afternoon napping information and cognition test scores for at least one wave, and completed the four times follow-up (*n* = 4,024). Written informed consent was provided by each participant, and CHARLS received ethical approval from the Peking University Institutional Review Board.

### Measurement

#### Napping

During wave1- wave4, afternoon napping duration was self-reported in face-to-face interviews without any given categories. Participants were asked to respond to the question “During the past month, how long did you take a nap after lunch (average minutes for 1 day)?” According to previous studies, we categorized afternoon napping into four groups: No napping, 0 min per day; Short napping, 0–30 min per day (not included 0 min); Moderate napping, 30–90 min per day (not included 30 min); Long napping, >90 min per day. According to Supplementary Figure 1, older adults with moderate napping had higher global cognition scores than other groups, and there is an m-shaped association between baseline napping and longitudinal global cognition scores. For participants with moderate napping at baseline were divided into five variation types: no change, decreased >0.5 h, increased >0.5 h, decreased 0–0.5 h, and increased 0–0.5 h. By using these divisions, we could explore changes in moderate napping and global cognition scores in different waves. According to the change in napping data at wave1-wave4, we defined the changes in napping for participants with no, short, and long napping duration at baseline into five categories: excessive, no change, benefit 1, benefit 2, and benefit 3 (specific definitions in the note of **Table 5**).

#### Cognition Assessments

Cognitive assessment was conducted in every wave and included memory, executive function, and orientation. The memory assessment test is comprised of the delayed and immediate word recall for 10 unrelated words. The memory score was the sum of words that were successfully recollected in the delayed and immediate word recall tasks, separately, ranging from 0 to 20. The orientation test comprised four questions regarding the date of the month, the day of the week, the month, and the year. Each correct answer counted one point. The executive function task was comprised of the serial sevens test, in which the respondent counts backward from 100 in increments of 7 (five successive counts, with one point given for each correct answer), and the intersecting pentagons copying test, in which the participants need to observe and draw a picture of two overlapping pentagons (three points were given for a successful drawing and none for an unsuccessful drawing). The executive score ranged from 0 to 8. The global cognition score was the sum of memory, executive function, and orientation scores, ranging from 0 to 32. Both the reliability and the validity of these tests have been well-documented (22, 23).

#### Co-variates Assessments

Potential confounders at baseline based on previous studies requiring adjustment were demographic factors, depression, chronic disease history, lifestyle behaviors, and taking tranquilizers or sleeping pills. Night sleep duration was self-reported by face-to-face interviews at each wave. Participants were asked to respond to the question “During the past month, how many hours of actual sleep did you get at night (average hours for one night)?” Ten-item Center for Epidemiologic Studies Depression Scale short form (CES-D) was used to evaluate participants' depressive symptoms (24). Each item was scored from 0 (rarely or none of the time) to 3 (most or all the time), and the total score was 30. People with a score of 12 or higher were assigned to the depressive group. Participants were asked to answer the highest level of education completed, and participants with the highest educational level under high school were divided into the low level of education group, conversely, their inclusion in the high educational level group. The history of chronic diseases collected by asking “Have you been diagnosed with a chronic disease (for example, hypertension) by a doctor?” Other details of the covariates are included in Supplementary Table 1.

### Statistical Analysis

The missing data were handled with multiple imputations. Spearman's Co-efficients were used to examine the correlations between napping duration and global cognition scores at four waves. The Analysis of Variance (ANOVA) was used to compare characteristics differences within napping duration groups at baseline. The longitudinal association between baseline afternoon napping and cognitive performance over 8 years was tested by using Generalized additive models (GAMs) and Generalized estimating equations (GEE). GEE could examine between-person variation and within-person correlation of repeated outcomes and GEE is suitable for dealing with repeated measurements data from a wide range of distribution types and addresses the issue of correlation in repeated measurements data. We calculated the association between baseline afternoon napping and longitudinal global cognition by adjusting models 1–2. Besides, we defined the participants into two groups according to their afternoon napping at baseline: napping ≤1.5 h in wave 1 and napping >1.5 h in wave 1. We also applied adjusted GEEs to test the linear trend association between baseline afternoon napping and longitudinal global cognition by using the duration of the afternoon napping in the two groups, separately. Since previous studies presented that the association between night sleep duration and longitudinal global cognition was not linear, the relationship between napping and longitudinal cognitive development may also not be linear, so we chose a GAM to explore the relationship between napping and long-term cognitive performance since GAMs allow us to fit the model using a non-linear smoothing term without prior knowledge of the relationship between the dependent and independent variables. GAMs were applied to examine the association between baseline afternoon napping and cognitive performance at wave 4 by fitting a smooth spline curve. Generalized linear models (GLMs) were used to analyze relationships between changes in afternoon napping and cognitive performance by adjusted models 1–3. For participants with no, short, and long napping duration at baseline, we conducted GLMs to test the associations between changes in napping defined before and cognitive performance in wave 4, separately. However, for participants with moderate napping at baseline, we applied several GLMs to examine the relationships between changes in napping and cognitive performance on each wave. We applied GLMs to examine the associations between changes in napping between wave 1 and wave 2 and global cognitive scores in wave 2, and similar steps were conducted on data between wave 1 and wave 3, wave 1 and wave 4. We conducted a GEE which was stratified by gender to test the sensitivity. All statistical analyses were tested by SPSS 22.0 and R3.5.1. Two-tail *P* values < 0.05 were considered statistically significant.

## Results

### Descriptive Statistics

After individuals without data on afternoon napping and cognitive performances were excluded, 4,024 participants were included in this study. The demographic and health characteristics of participants at baseline are shown in Table 1. The sample included 4,024 individuals over 60 years old at baseline (48.4% male), the average global cognition score was 12.37 (SD: 6.34) at baseline, and the average self-reported afternoon napping duration was 0.54 (SD: 0.71) h per day at baseline. At baseline, 16.7% of the individuals were no nappers, 25.6% were short nappers, 12.0% were moderate nappers, and 45.7% were long nappers. Participants with moderate afternoon napping were more likely to be males, to have slight BMI, to have shorter night sleep duration, and to be smokers at baseline.

**Table 1:** Demographic and health characteristics of the study population at baseline.

	**Napping duration groups (categorized by sleep daytime napping in Wave 1)**				
	**0 min (670)**	**0–30 min (1,032)**	**30–90 min (481)**	**>90 min (1,841)**	**All (4,024)**	***P*-value**
**Continuous variables, mean (SD)**						
Age, years	66.70 (5.79)	66.73 (5.77)	67.21 (6.05)	67.23 (6.11)	66.90 (5.89)	0.069
BMI, kg/m^2^	22.72 (4.09)	23.28 (4.20)	23.27 (3.79)	23.31 (3.84)	23.02 (4.01)	**<0.001**
Sleep duration per night, hours	5.94 (2.07)	6.11 (1.91)	5.34 (1.81)	6.72 (1.94)	6.16 (1.98)	**<0.001**
Baseline cognition score	11.80 (6.31)	13.20 (6.12)	12.70 (6.56)	12.70 (6.10)	12.37 (6.34)	**<0.001**
**Categorical variables**, ***n*** **(%)**						
Gender (male)	316 (47.2)	592 (57.4)	290 (60.3)	751 (40.8)	1,949 (48.4)	**<0.001**
High level of education	36 (5.4)	70 (6.8)	26 (5.4)	73 (4.0)	205 (5.1)	**0.011**
Physical activity						
Vigorous physical activities	140 (20.9)	226 (21.9)	97 (20.2)	444 (24.1)	907 (22.5)	0.141
Moderate physical activities	185 (27.6)	277 (26.8)	116 (24.1)	531 (28.8)	1,109 (27.6)	0.200
Walking	316 (47.2)	460 (44.6)	183 (38.0)	855 (46.4)	1,814 (45.1)	**0.006**
Depression symptoms	350 (52.2)	523 (50.7)	234 (48.6)	894 (48.6)	2,001 (49.7)	0.349
Taking sleeping pills	15 (2.2)	14 (1.4)	10 (2.1)	23 (1.2)	62 (1.5)	0.228
Current smoking	341 (50.9)	559 (54.2)	282 (58.6)	1,045 (56.8)	2,227 (55.3)	**0.023**
Current alcohol use	529 (79.0)	307 (29.7)	134 (27.9)	383 (20.8)	965 (24.0)	**<0.001**
Medical history						
Hypertension	208 (31.0)	334 (32.4)	154 (32.0)	462 (25.1)	1,158 (28.8)	**<0.001**
Dyslipidemia	80 (11.9)	140 (13.6)	54 (11.2)	135 (7.3)	409 (10.2)	**<0.001**
Diabetes	67 (10.0)	81 (7.8)	27 (5.6)	72 (3.9)	247 (6.1)	**<0.001**
Cancer	8 (1.2)	10 (1.0)	1 (0.2)	13 (0.7)	32 (0.8)	0.258
Chronic lung diseases	79 (11.8)	155 (15.0)	67 (13.9)	210 (11.4)	511 (12.7)	**0.030**
Liver disease	25 (3.7)	40 (3.9)	19 (4.0)	72 (3.9)	156 (3.9)	0.997
Heart problems	117 (17.5)	166 (16.1)	70 (14.6)	229 (12.4)	582 (14.5)	**0.004**
Stroke	23 (3.4)	35 (3.4)	15 (3.1)	36 (2.0)	109 (2.7)	0.059
Kidney disease	48 (7.2)	69 (6.7)	25 (5.2)	123 (6.7)	265 (6.6)	0.590
Stomach or other digestive disease	157 (23.4)	242 (23.4)	117 (24.3)	456 (24.8)	972 (24.2)	0.836
Emotional, nervous, or psychiatric problems	15 (2.2)	13 (1.3)	9 (1.9)	22 (1.2)	59 (1.5)	0.207
Memory-related disease	11 (1.6)	20 (1.9)	14 (2.9)	29 (1.6)	74 (1.8)	0.265
Arthritis or rheumatism	271 (40.4)	382 (37.0)	182 (37.8)	759 (41.2)	1,594 (39.6)	0.125
Asthma	29 (4.3)	54 (5.2)	31 (6.4)	86 (4.7)	200 (5.0)	0.348

*BMI, body mass index. The bold values provided in table means that P < 0.05*.

Supplementary Table 2 shows the correlations between afternoon napping and global cognition scores. There were positive relationships between cognitive performances and napping durations during four waves. To examine the longitudinal association between baseline napping and cognitive performance at wave 4 and the associations between the changes in napping and cognitive performance at wave 4, further analyses were conducted.

### Associations Between Afternoon Napping and Longitudinal Cognitive Performance

The results of GEE which examined the associations between baseline afternoon napping and longitudinal global cognition are presented in Table 2. The results of GEE showed the relationship between baseline daytime napping and longitudinal cognition domains (orientation, memory, and executive function) are shown in Table 3. Individuals with afternoon napping in wave 1 had better global cognition scores than the person who did not have afternoon napping at wave 1, as well as better scores in specific domains orientation and executive function. To be more specific, participants with short afternoon napping at baseline had better global cognition scores than people without napping and had higher scores in all three aspects. At the same time, people who had moderate afternoon napping also had higher global cognition scores due to the data from adjusted model 1.

**Table 2:** Associations between baseline daytime napping and longitudinal cognitive function according to generalized estimating equations (GEE).

	**Model 1^**a**^ β (SE)**	**Model 2^**b**^ β (SE)**
**Categorical trend 1**		
No napping	Ref.	Ref.
Short napping	1.248 (0.773, 1.723)^***^	0.901 (0.495, 1.344)^***^
Moderate napping	0.772 (0.376, 1.169)^***^	0.327 (−0.053, 0.707)
Long napping	0.256 (−0.251, 0.763)	−0.032 (−0.519, 0.455)
**Categorical trend 2**		
No napping	Ref.	Ref.
Napping	0.810 (0.486, 1.134)^***^	0.435 (0.125, 0.745)^**^
**Linear trend for napping** **≤1.5 h**		
Duration	0.642 (0.281, 1.004)^***^	0.218 (−0.130, 0.566)
**Linear trend for napping** **>1.5 h**		
Duration	−1.603 (−3.026, −0.180)^*^	−1.208 (−2.616, 0.201)

*** *P < 0.001*,

** *P < 0.01*,

* *P < 0.05, β: unstandardized beta Co-efficient, SE: standard error*.

a *Adjusted for age and sex*.

b *Adjusted for age, sex, educational levels, physical activities, BMI, depression, use of tranquilizers, smoking, alcohol consumption, night sleep duration at baseline, medical history including hypertension, dyslipidemia, heart disease, and other 11 chronic diseases*.

**Table 3:** Associations between baseline daytime napping and longitudinal cognitive function according to generalized estimating equations (GEE).

	**Orientation**	
	**Model 1^**a**^ β (SE)**	**Model 2^**b**^ β (SE)**
**Categorical trend 1**		
No napping	Ref.	Ref.
Short napping	0.251 (0.160, 0.343)^***^	0.195 (0.107, 0.282)^***^
Moderate napping	0.145 (0.065, 0.225)^***^	0.077 (−0.001, 0.155)
Long napping	0 (−0.104, 0.105)	−0.043 (−0.146, 0.059)
**Categorical trend 2**		
No napping	Ref.	Ref.
Napping	0.147 (0.082, 0.212)^***^	0.089 (0.025, 0.153)^**^
**Linear trend for napping** **≤1.5 h**		
Duration	0.120 (0.048, 0.193)^***^	0.055 (−0.017, 0.127)
**Linear trend for napping** **>1.5 h**		
Duration	−0.313 (−0.640, 0.014)	−0.211 (−0.540, 0.118)
**Memory**		
**Categorical trend 1**		
No napping	Ref.	Ref.
Short napping	0.550 (0.303, 0.796)^***^	0.385 (0.153, 0.618)^***^
Moderate napping	0.314 (0.105, 0.522)^***^	0.111 (−0.091, 0.313)
Long napping	0.061 (−0.207, 0.329)	−0.057 (−0.315, 0.201)
**Categorical trend 2**		
No napping	Ref.	Ref.
Napping	0.333 (0.163, 0.503)^***^	0.163 (−0.001, 0.328)
**Linear trend for napping** **≤1.5 h**		
Duration	0.242 (0.052, 0.432)^*^	0.049 (−0.135, 0.233)
**Linear trend for napping** **>1.5 h**		
Duration	−0.499 (−1.220, 0.222)	−0.245 (−0.980, 0.490)
**Executive function**		
**Categorical trend 1**		
No napping	Ref.	Ref.
Short napping	0.447 (0.249, 0.645)^***^	0.321 (0.133, 0.510)^***^
Moderate napping	0.314 (0.149, 0.479)^***^	0.139 (−0.021, 0.300)
Long napping	0.195 (−0.016, 0.405)	0.069 (−0.138, 0.275)
**Categorical trend 2**		
No napping	Ref.	Ref.
Napping	0.330 (0.194, 0.465)^***^	0.183 (0.051, 0.314)^**^
**Linear trend for napping** **≤1.5 h**		
Duration	0.279 (0.128, 0.431)^***^	0.114 (−0.034, 0.262)
**Linear trend for napping** **>1.5 h**		
Duration	−0.791 (−1.387, −0.195)^**^	−0.752 (−1.322, −0.182)^*^

*** *P < 0.001*,

** *P < 0.01*,

* *P < 0.05, β: unstandardized beta Co-efficient, SE: standard error*.

a *Adjusted for age and sex*.

b *Adjusted for age, sex, educational levels, physical activities, BMI, depression, use of tranquilizers, smoking, alcohol consumption, night sleep duration at baseline, medical history including hypertension, dyslipidemia, heart disease, and other 11 chronic diseases*.

Linear trends were significant due to the data of adjusted model 1. For participants whose napping was ≤1.5 h in wave 1, a shorter napping duration was associated with a lower global cognition score and the same results in all three domains, but for participants with napping >1.5 h in wave 1, a longer napping duration was related to lower global cognition score, to be more specific, longer napping duration was associated with lower executive function scores.

Supplementary Figure 1 showed the relationships between afternoon napping at wave 1 and cognitive performance at wave 4 with a non-linear curve. Supplementary Figures 1A–C visualized the results of GAM for global cognition score in wave 4. The EDF values presented a non-linear fit, and the plots indicated an inconspicuous M-shape association between afternoon napping and global cognition score in both univariable and multivariable GAMs. The highest global cognition scores at wave 4 appeared among those who had nearly 0.5- or 1.5-h afternoon napping at baseline according to the plots.

### Associations Between Changes in Napping and Longitudinal Cognitive Performance

#### Participants With Moderate Afternoon Napping in Wave 1

The effects of changes in afternoon napping on global cognition scores at wave 2, wave 3, and wave 4 among participants with moderate afternoon napping were shown in Table 4, the descriptive results of GLMs are included in Supplementary Table 3. For people with 30–90 min of afternoon napping per day, napping decreased by >30 min per day from wave 1 to wave 2 was associated with worse cognitive performance than those who remained in the moderate afternoon napping group according to Model 1 and Model 2. For participants with moderate napping, napping decreased or increased by >30 min per day from 2011 to 2018 and was related to worse cognitive function than those of the no-change group according to Model 1. However, the results of Model 2 and Model 3 showed that napping increased by >30 min per day from 2011 to 2018 was related to worse cognitive function than those of the no-change group.

**Table 4:** Associations between changes in daytime napping and global cognition among participants whose napping durations were 30–90 min at baseline^a^.

	**Unstandardized beta Co-efficient (95% confidence interval)**				
	**Type of changes in napping** ^**b**^				
	**Decreased by >0.5 h**	**Decreased by 0–0.5 h**	**No change**	**Increased by 0–0.5 h**	**Increased by >0.5 h**
**Global cognition in wave 2^b^**
Model 1	−1.587 (−2.583, −0.590)^**^	−1.023 (−2.223, 0.177)	Ref.	−1.164 (−2.457, 0.129)	−0.996 (−2.183, 0.191)
Model 2	−1.108 (−2.072, −0.144)^*^	−0.688 (−1.842, 0.466)	Ref.	−0.968 (−2.209, 0.273)	−0.625 (−1.767, 0.517)
Model 3	−0.769 (−1.600, 0.062)	−0.531 (−1.525, 0.463)	Ref.	−0.824 (−1.893, 0.245)	−0.253 (−1.237, 0.732)
**Global cognition in wave 3^c^**
Model 1	−0.824 (−1.767, 0.120)	−0.226 (−1.372, 0.920)	Ref.	0.070 (−1.118, 1.257)	−0.927 (−2.021, 0.166)
Model 2	−0.445 (−1.364, 0.474)	−0.022 (−1.137, 1.093)	Ref.	0.301 (−0.849, 1.452)	−0.396 (−1.464, 0.672)
Model 3	−0.128 (−0.911, 0.655)	−0.098 (−1.047, 0.852)	Ref.	0.364 (−0.616, 1.343)	−0.019 (−0.930, 0.891)
**Global cognition in wave 4^d^**
Model 1	−1.210 (−2.298, −0.121)^***^	1.328 (−0.040, 2.696)	Ref.	0.268 (−1.152, 1.688)	−1.952 (−3.183, −0.722)^**^
Model 2	−0.897 (−1.952, 0.158)	1.198 (−0.114, 2.510)	Ref.	0.385 (−0.980, 1.751)	−1.558 (−2.738, −0.379)^*^
Model 3	−0.147 (−1.060, 0.766)	1.002 (−0.129, 2.134)	Ref.	0.303 (−0.874, 1.480)	−1.123 (−2.141, −0.105)^*^

a *Using generalized linear models (GLMs)*.

* *P < 0.05*,

** *P < 0.01*,

*** *P < 0.001*.

*Model 1: Adjusted for age and gender*.

*Model 2: Adjusted for Model 1+ educational levels, physical activities, BMI, depression, use of tranquilizers, smoking, alcohol consumption, night sleep duration at baseline, medical history including hypertension, dyslipidemia, heart disease, and other 11 chronic diseases*.

*Model 3: Adjusted for Model 2 + baseline global cognition score*.

b *Change in napping presented the variations between napping in wave 2 and napping at baseline, being divided into five groups: decreased >0.5 h/day, decrease 0–0.5 h/day, no change, increased 0–0.5 h/day, and increased >0.5 h/day, the GLMs showed the associations between this change in napping and global cognition scores in wave 2*.

c *Change in napping presented the variations between napping in wave 3 and napping at baseline, being divided into five groups: decreased >0.5 h/day, decrease 0–0.5 h/day, no change, increased 0–0.5 h/day, and increased >0.5 h/day, the GLMs showed the associations between this change in napping and global cognition scores in wave 3*.

d *Change in napping presented the variations between napping in wave 4 and napping at baseline, being divided into five groups: decreased >0.5 h/day, decrease 0–0.5 h/day, no change, increased 0–0.5 h/day, and increased >0.5 h/day, the GLMs showed the associations between this change in napping and global cognition scores in wave 4*.

#### Participants Without Afternoon Napping, With Short or Long Afternoon Napping in Wave 1

The effects of napping changes on participants who were not in the moderate-napping group in wave 1 were studied by subgroup analyses in Table 5, and the characteristics of naptime variation grouping are presented in Supplementary Table 4. Compared with the “No change” group, the “Excessive” group had worse cognitive performance in wave 4 among participants without napping at baseline according to the results of Model 2. For participants with short afternoon napping in wave 1, people in the “Excessive” group had lower global cognition scores in wave 4 than the “No change” group according to the results of Model 1 and Model 2. However, after adjusted baseline global cognition scores, there is no significant association found between changes in napping from wave 1 to wave 4 and global cognition scores in wave 4.

**Table 5:** Unstandardized β Co-efficients for adjusted generalized linear models examining the relationships between variations in daytime napping and global cognition at wave 4 among participants whose napping = 0, <30 min or >90 min at baseline^a^.

	**Unstandardized beta Co-efficient (95% confidence interval)**				
	**Type of changes in napping^b^**				
	**Excessive^c^**	**No change^d^**	**Benefit 1^e^**	**Benefit 2^f^**	**Benefit 3^g^**
**No napping in wave 1**					
Model 1	−0.412 (−1.083, 0.259)	Ref.	−0.787 (−1.722, 0.148)	0.930 (−0.483, 2.342)	−0.884 (−2.804, 1.035)
Model 2	−0.694 (−1.335, −0.053)^*^	Ref.	−0.764 (−1.654, 0.125)	0.462 (−0.885, 1.809)	−0.786 (−2.609, 1.038)
Model 3	−0.447 (−1.001, 0.107)	Ref.	−0.537 (−1.305, 0.231)	0.181 (−0.982, 1.334)	−0.670 (−2.245, 0.904)
**Short napping in wave 1**					
Model 1	−3.712 (−5.977, −1.447)^***^	Ref.	1.903 (−1.097, 4.902)	−2.568 (−5.282, 0.146)	0.803 (−2.010, 3.616)
Model 2	−2.593 (−4.763, −0.423)^*^	Ref.	1.067 (−1.785, 3.919)	−1.990 (−4.588, 0.607)	0.806 (−1.863, 3.475)
Model 3	−1.391 (−3.245, 0.464)	Ref.	1.602 (−0.828, 4.033)	−0.795 (−3.013, 1.423)	1.609 (−0.666, 3.885)
**Long napping in wave 1**					
Model 1	−1.246 (−3.024, 0.533)	Ref.	−0.737 (−2.956, 2.483)	0.560 (−2.032, 3.151)	0.135 (−2.879, 3.149)
Model 2	−1.556 (−3.270, 0.158)	Ref.	−0.744 (−2.895, 1.408)	0.218 (−2.275, 2.710)	−0.195 (−3.091, 2.702)
Model 3	−0.863 (−2.374, 0.647)	Ref.	−0.427 (−2.319, 1.464)	0.868 (−1.325, 3.061)	0.480 (−2.068, 3.027)

a ^*^ P < 0.05, ^**^ P < 0.01, ^***^ P < 0.001.

*Model 1: Adjusted for age and gender*.

*Model 2: Adjusted for Model 1+ educational levels, physical activities, BMI, depression, use of tranquilizers, smoking, alcohol consumption, night sleep duration at baseline, medical history including hypertension, dyslipidemia, heart disease, and other 11 chronic diseases*.

*Model 3: Adjusted for Model 2 + baseline global cognition score*.

b *Type of napping's variation was decided by patterns of daytime napping in Wave 2–Wave 4*.

c *Excessive, for participants with No napping in Wave 1, they were divided into Excessive group as long as they were in No or Long napping group once from Wave 2 to Wave 4; for participants with Short napping at baseline, they were divided into Excessive group as long as they were in No or Long napping group once from Wave 2 to Wave 4; for participants with Long napping at baseline, they were divided into Excessive group as long as they were in No or Long napping group once from Wave 2 to Wave 4*.

d *No change, for participants with No napping in Wave 1, they were divided into No change group if they maintained in No napping group from Wave 2 to Wave 4; for participants with Short napping at baseline, they were divided into No change group if they maintained in Short napping group once from Wave 2 to Wave 4; for participants with Long napping at baseline, they were divided into No change group if they maintained in Long napping group once from Wave 2 to Wave 4*.

e *Benefit 1, for participants with No napping in Wave 1, they were divided into Benefit 1 group if they maintained No napping in two waves and had an Moderate napping in another wave; for participants with Short napping at baseline, they were divided into Benefit 1 group if they maintained Short napping in two waves and had an Moderate napping in another wave; for participants with Long napping at baseline, they were divided into Benefit 1 group if they maintained an Long napping in two waves and had an Moderate napping in another wave*.

f *Benefit 2, for participants with No napping in Wave 1, they were divided into Benefit 2 group if they maintained in No napping group for one wave and in Moderate napping group for other two waves; for participants with Short napping at baseline, they were divided into Benefit 2 group if they maintained an Short napping in one wave and had two Moderate napping in other two waves; for participants with Long napping at baseline, they were divided into Benefit 2 group if they maintained an Long napping in one wave and had two Moderate napping in other two waves*.

g *Benefit 3, for participants with No napping in Wave 1, they were divided into Benefit 3 group if they maintained in Moderate napping in three waves; for participants with Short napping at baseline, they were divided into Benefit 3 group if they Moderate napping in three waves; for participants with Long napping at baseline, they were divided into Benefit 3 group if they Moderate napping in three waves*.

### Sensitivity Analyses

Separate analyses were performed by gender to assess the potential differences between male and female participants on the main results. The results showed similar associations between baseline afternoon napping and cognitive function in females. However, these associations were no longer statistically significant for males (Supplementary Table 3).

## Discussion

In this study, we observed an m-shaped association between baseline afternoon napping and global cognition over 8 years among Chinese old people. For moderate nappers, a >30 min change in afternoon napping was significantly associated with worse cognitive function. Compared to those whose napping duration remained unchanged, a move from no napping to long/short napping was related to lower global cognition, and a move from short napping to long/no napping was also associated with worse global cognition. A change from no napping, short napping, or long napping to moderate napping had no statistically significant effect.

The results showed that participants with afternoon napping at baseline would have better cognitive performance, which is along with a study reported that napping was associated with a decreased risk of cognitive decline after both 2 and 10 years (25). To be more specific, old adults with short napping would have higher cognition scores than participants without baseline afternoon napping. A recent longitudinal study (26) also indicated that afternoon napping for fewer than 30 min benefited the cognitive function, in which afternoon napping over 120 min per day was more likely to develop cognitive impairment, which is along with our results that a longer napping duration was associated with lower global cognition score among participants with napping >90 min in wave 1. The underlying mechanism of which short napping's benefits is unclear, inflammation (27) and clearing of brain β-amyloid (28) were possible conjectures. Short napping could be of advantage in clearing brain β-amyloid by improving sleep quality since the low sleep quality is associated with brain β-amyloid burden among old adults (28). Sleep participants in the sympathetic nervous system and hypothalamus-pituitary-adrenal axis which changes the basal gene expression profile and leads to more proinflammatory skewing (29, 30). But afternoon napping is thought to be an evolved response to inflammation in particular (31). Meanwhile, napping might be neuroprotective and could slow the development of vascular and neurodegenerative pathologies, by reducing oxidative stress (32) or lowering vascular demands (33).

Changes in afternoon napping had different effects on cognitive performance for 8 years for no, short, moderate, and long nappers at baseline. For moderate nappers, a >30 min change means a move from moderate napping to extreme napping, as the previous cross-sectional study proved that, was related to an increased risk of cognitive decline (26). The possible underlying mechanism is related to altered circadian rhythm. Previous studies proved the benefit of napping on the sleep deprivation from the former night, a change in napping could reflect the change in circadian rhythm for every 24 h (34), and a bidirectional relationship between sleep duration, circadian rhythm, and cognitive function was proved by the previous analysis (35). Due to some studies, the sleep-related circadian rhythm was regarded as a risk factor associated with neurodegenerative diseases and an altered factor related to neurodegeneration, including clearance of toxic proteins, neuroinflammation, and Aβ dynamics (36). The results in the study showed that, compared with Model 2's results with adjustments of potential covariates, after adjusted potential covariates and baseline global cognition scores, moderate nappers with an increase of >30 min napping per day had lower global cognition scores in wave 4, and other changes in napping did not significantly associate with later global cognition scores. These suggest that there might be reverse causality in baseline cognition and afternoon napping. Baseline cognition could influence older adults' afternoon napping and cause the individuals' afternoon napping to increase or decrease in the following years. More studies with stronger causal arguments are needed in the future to infer whether there is a reverse causal relationship between napping and cognition in the future.

For short nappers, a move to no or long napping presented an increased risk of lower global cognition than the “no change” group. Previous studies proved that no napping or long napping had detrimental effects on cognitive performance, and short napping (<30 min per day) was a benefit for people's cognitive function (13, 25). Old adults who moved from short napping to no or long napping could be defined as a high-risk group for cognitive decline and cognitive impairment. When adjusted baseline global cognition scores, no significant association was found in Model 3. This is probably because the reverse causality existed between afternoon napping and baseline cognition among short nappers.

However, for long nappers, no significant difference was found in the effect of change in afternoon napping. People who consciously took long afternoon napping could represent a group of people with inflammation or chronic disease and unhealthy habits, who need long napping for recovering from the bad condition of their bodies. It is possible that long daytime napping is a risk marker that could reflect underlying health conditions that impact cognition. In this situation, reducing the napping duration would not be a beneficial way for them (27, 37). Meanwhile, Leng et al. (26) reported that excessive napping could be considered a robust independent early risk marker of future cognitive decline and impairment in elderly persons. The “long nappers” might be phenotyped as a “hyper somnolent” group genetically who are with more likelihood of falling asleep, so they would have higher sleep efficiency and relatively long sleep duration. This group might have common pathways to neuropsychiatric conditions at the same time.

### Strength and Limitations

To our knowledge, this is the first study to test the longitudinal association between baseline napping, change in napping, and cognitive performance over 8 years, and our analysis used a large, longitudinal, and representative sample. Neurocognitive tasks used to test participants' cognitive performance were the same for every wave, which facilitated the comparing analysis across time. The robustness of the cohort with enough follow-up years and multiple confounders allows us to observe how the longitudinal relationships with adjustments.

Our study had several limitations. The results could have a selective bias since we excluded participants without finishing all the follow-ups, which could limit the representativeness of the present results. Afternoon napping was assessed by self-reported analyses in this study, and this could still cause inaccuracies by recall bias compared to objective afternoon napping durations. In our analysis, we assumed that afternoon napping which people self-reported was planned, not unconscious excessive daytime sleepiness among the old participants. However, the naps reported in our sample could be resulted from other conditions, such as excessive daytime sleepiness and sleep disturbance. We cannot adjust these possible confounders according to the absence of the measurements in CHARLS. Although we adjusted for numerous confounders in our analyses, unmeasured covariates including napping frequency and whether naps are scheduled naps might still lead to confounding bias. Besides, many potential covariates such as what kind of physical diseases participants had in the study were from subjective reports of respondents, which could result in recall bias or reporting bias. Meanwhile, the cognitive development information was tested by idiosyncratic combinations of validated cognitive tasks instead of a standardized cognitive battery, which could also result in biases. Reverse causality may exist between cognition and afternoon napping, further study with stronger causal arguments.

## Conclusion

In conclusion, using data from a longitudinal study of large groups across multiple time points, we reaffirmed that napping could be a marker of cognition change. We observed that adults over 60 years old with napping <30 min per day may be at lower risk of cognitive decline. Old people who have changes in napping, especially a move to extreme napping, would be defined as a high-risk group of cognitive decline and cognitive impairment. This finding will be useful for doctors to provide daytime napping recommendations for old people and help clinicians to identify the old people at risk. Further prospective experimental studies with objective measurements of daytime napping are needed for exploring the effects of daytime napping on cognition and providing specific interventions with napping protocols for old adults.

## Data Availability Statement

Publicly available datasets were analyzed in this study. This data can be found here: http://charls.pku.edu.cn/.

## Ethics Statement

The studies involving human participants were reviewed and approved by Peking University Institutional Review Board. The participants provided their written informed consent to participate in this study.

## Author Contributions

CL conceptualized and designed the study, developed the data extraction instrument, collected data and carried out the initial analysis, drafted, and revised the manuscript. YY conceptualized the study, supervised data collection, and critically screened important intellectual contents of the manuscript. Both authors have read and approved the manuscript as submitted and agree to be accountable for all aspects of the works.

## Funding

This work was funded by the National Natural Science Foundations (NSFC) of China [grant numbers 81973153, 81373101, and 81673276] and the Central South University [grant number 2021zzts0965]. The data collection was supported by the Behavioral and Social Research Division of the National Institute on Ageing of the National Institute of Health (grants 1-R21-AG031372-01, 1-R01-AG037031-01, and 3-R01AG037031-03S1), the Natural Science Foundation of China (grants 70773002, 70910107022, and 71130002), and the World Bank (contracts 7145915 and 7159234), and Peking University.

## Conflict of Interest

The authors declare that the research was conducted in the absence of any commercial or financial relationships that could be construed as a potential conflict of interest.

## Publisher's Note

All claims expressed in this article are solely those of the authors and do not necessarily represent those of their affiliated organizations, or those of the publisher, the editors and the reviewers. Any product that may be evaluated in this article, or claim that may be made by its manufacturer, is not guaranteed or endorsed by the publisher.

## References

[1] Prince M Bryce R Albanese E Wimo A Ribeiro W Ferri CP. The global prevalence of dementia: a systematic review and meta analysis. Alzheimer's Dementia. (2013) 9:63–75.e2. 10.1016/j.jalz.2012.11.007 23305823

[2] World Health Organization Alzheimer's Disease International. Dementia: A Public Health Priority. Geneva: World Health Organization (2012).

[3] National Bureau of Statistics of the People's Republic of China. The Main Data Announcement of Sixth National Census, 2011. Beijing: National Bureau of Statistics of the People's Republic of China (2017).

[4] Hebert LE Weuve J Scherr PA Evans DA. Alzheimer's disease in the United States (2010–2050) estimated using the 2010 census. Neurology. (2013) 80:1778–83. 10.1212/WNL.0b013e31828726f5 23390181 PMC3719424

[5] Alzheimer's disease facts and figures. Alzheimer's Dementia. (2021) 17:327–406. 10.1002/alz.12328 33756057

[6] Jia J Wei C Chen S Li F Tang Y Qin W. The cost of Alzheimer's disease in China and re-estimation of costs worldwide. Alzheimers Dement. (2018) 14:483–91. 10.1016/j.jalz.2017.12.006 29433981

[7] Livingston G Sommerlad A Orgeta V Costafreda SG Huntley J Ames D. Dementia prevention, intervention, and care. Lancet. (2017) 390:2673–734. 10.1016/S0140-6736(17)31363-6 28735855

[8] Westwood AJ Beiser A Jain N Himali JJ DeCarli C Auerbach SH. Prolonged sleep duration as a marker of early neurodegeneration predicting incident dementia. Neurology. (2017) 88:1172–9. 10.1212/WNL.0000000000003732 28228567 PMC5373785

[9] Blackwell T Yaffe K Ancoli-Israel S Redline S Ensrud KE Stefanick ML. Association of sleep characteristics and cognition in older community-dwelling men: the MrOS sleep study. Sleep. (2011) 34:1347–56. 10.5665/SLEEP.1276 21966066 PMC3174836

[10] Li J Chang YP Porock D. Factors associated with daytime sleep in nursing home residents. Res Aging. (2015) 37:103–17. 10.1177/0164027514537081 25651553

[11] Alzheimer's disease facts and figures. Alzheimer's Dementia. (2015) 11:332–84. 10.1016/j.jalz.2015.02.003 25984581

[12] Fang W Li Z Wu L Cao Z Liang Y Yang H. Longer habitual afternoon napping is associated with a higher risk for impaired fasting plasma glucose and diabetes mellitus in older adults: results from the Dongfeng-Tongji cohort of retired workers. Sleep Med. (2013) 14:950–4. 10.1016/j.sleep.2013.04.015 23831240

[13] Owusu JT Wennberg AMV Holingue CB Tzuang M Abeson KD Spira AP. Napping characteristics and cognitive performance in older adults. Int J Geriatr Psychiatry. (2019) 34:87–96. 10.1002/gps.4991 30311961 PMC6445640

[14] Lin JF Li FD Chen XG He F Zhai YJ Pan XQ. Association of post-lunch napping duration and night-time sleep duration with cognitive impairment in Chinese elderly: a cross-sectional study. BMJ Open. (2018) 8:e023188. 10.1136/bmjopen-2018-023188 30552262 PMC6303738

[15] Li J Chang YP Riegel B Keenan BT Varrasse M Pack AI. Intermediate, but not extended, afternoon naps may preserve cognition in Chinese older adults. J Gerontol A Biol Sci Med Sci. (2018) 73:360–6. 10.1093/gerona/glx069 28475689 PMC5861921

[16] Li J Cacchione PZ Hodgson N Riegel B Keenan BT Scharf MT. Afternoon napping and cognition in Chinese older adults: findings from the China health and retirement longitudinal study baseline assessment. J Am Geriatr Soc. (2017) 65:373–80. 10.1111/jgs.14368 27995615 PMC6487643

[17] Xin C Zhang B Fang S Zhou J. Daytime napping and successful aging among older adults in China: a cross-sectional study. BMC Geriatr. (2020) 20:2. 10.1186/s12877-019-1408-4 31898552 PMC6941277

[18] Zhao X Cheng L Zhu C Cen S Lin W Zheng W. A double-edged sword: the association of daytime napping duration and metabolism related diseases in a Chinese population. Eur J Clin Nutr. (2021) 75:291–8. 10.1038/s41430-020-00777-2 33082534

[19] Cai H Su N Li W Li X Xiao S Sun L. Relationship between afternoon napping and cognitive function in the aging Chinese population. General Psychiatry. (2021) 34:e100361. 10.1136/gpsych-2020-100361 33585792 PMC7839842

[20] Zhao Y Hu Y Smith JP Strauss J Yang G. Cohort profile: the China health and retirement longitudinal study (CHARLS). Int J Epidemiol. (2014) 43:61–8. 10.1093/ije/dys203 23243115 PMC3937970

[21] Yaohui Z Strauss J Chen X Wang Y Gong J Meng Q. China Health and Retirement Longitudinal StudyWave 4 User's Guide. Beijing: Peking University (2020).

[22] Xie W Zheng F Yan L Zhong B. Cognitive decline before and after incident coronary events. J Am Coll Cardiol. (2019) 73:3041–50. 10.1016/j.jacc.2019.04.019 31221251

[23] Xu H Zhang Z Li L Liu J. Early life exposure to China's 1959–61 famine and midlife cognition. Int J Epidemiol. (2018) 47:109–20. 10.1093/ije/dyx222 29126190 PMC6075478

[24] Chen H Mui AC. Factorial validity of the center for epidemiologic studies depression scale short form in older population in China. Int Psychogeriatr. (2014) 26:49–57. 10.1017/S1041610213001701 24125553

[25] Keage HA Banks S Yang KL Morgan K Brayne C Matthews FE. What sleep characteristics predict cognitive decline in the elderly? Sleep Med. (2012) 13:886–92. 10.1016/j.sleep.2012.02.003 22560827

[26] Leng Y Redline S Stone KL Ancoli-Israel S Yaffe K. Objective napping, cognitive decline, and risk of cognitive impairment in older men. Alzheimer's Dementia. (2019) 15:1039–47. 10.1016/j.jalz.2019.04.009 31227429 PMC6699896

[27] Hu M Shu X Feng H Xiao LD. Sleep, inflammation and cognitive function in middle-aged and older adults: a population-based study. J Affect Disord. (2021) 284:120–5. 10.1016/j.jad.2021.02.013 33592430

[28] Cordone S Annarumma L Rossini PM De Gennaro L. Sleep and β-amyloid deposition in Alzheimer's disease: insights on mechanisms and possible innovative treatments. Front Pharmacol. (2019) 10:695. 10.3389/fphar.2019.00695 31281257 PMC6595048

[29] Irwin MR Cole SW. Reciprocal regulation of the neural and innate immune systems. Nat Rev Immunol. (2011) 11:625–32. 10.1038/nri3042 21818124 PMC3597082

[30] Irwin MR Olmstead R Carroll JE. Sleep disturbance, sleep duration, and inflammation: a systematic review and meta-analysis of cohort studies and experimental sleep deprivation. Biol Psychiatry. (2016) 80:40–52. 10.1016/j.biopsych.2015.05.014 26140821 PMC4666828

[31] Faraut B Nakib S Drogou C Elbaz M Sauvet F De Bandt JP. Napping reverses the salivary interleukin-6 and urinary norepinephrine changes induced by sleep restriction. J Clin Endocrinol Metab. (2015) 100:E416–26. 10.1210/jc.2014-2566 25668196

[32] Praticò D Clark CM Liun F Rokach J Lee VY Trojanowski JQ. Increase of brain oxidative stress in mild cognitive impairment: a possible predictor of Alzheimer's disease. Arch Neurol. (2002) 59:972–6. 10.1001/archneur.59.6.972 12056933

[33] Dufouil C de Kersaint-Gilly A Besançon V Levy C Auffray E Brunnereau L. Longitudinal study of blood pressure and white matter hyperintensities: the EVA MRI COHORT. Neurology. (2001) 56:921–6. 10.1212/WNL.56.7.921 11294930

[34] Auyeung TW Lee JS Leung J Kwok T Leung PC Woo J. Cognitive deficit is associated with phase advance of sleep-wake rhythm, daily napping, and prolonged sleep duration–a cross-sectional study in 2,947 community-dwelling older adults. Age. (2013) 35:479–86. 10.1007/s11357-011-9366-6 22215376 PMC3592949

[35] Wang C Holtzman DM. Bidirectional relationship between sleep and Alzheimer's disease: role of amyloid, tau, and other factors. Neuropsychopharmacology. (2020) 45:104–20. 10.1038/s41386-019-0478-5 31408876 PMC6879647

[36] Leng Y Musiek ES Hu K Cappuccio FP Yaffe K. Association between circadian rhythms and neurodegenerative diseases. Lancet Neurol. (2019) 18:307–18. 10.1016/S1474-4422(18)30461-7 30784558 PMC6426656

[37] Cross N Terpening Z Rogers NL Duffy SL Hickie IB Lewis SJ. Napping in older people “at risk” of dementia: relationships with depression, cognition, medical burden and sleep quality. J Sleep Res. (2015) 24:494–502. 10.1111/jsr.12313 26096839

