# Supplementary material for: Afternoon Napping Durations in Chinese Population Over 60 Years Old: Longitudinal Associations With Cognitive Performance
Source: Front Public Health. 2022 Jul 7;10:911498. doi: 10.3389/fpubh.2022.911498 (PMC9302202; doi:10.3389/fpubh.2022.911498)
Supplement: Supplementary file 1 [file Data_Sheet_1.pdf]

Supplemental files1

Supplemental Table 1. Definitions of variables used
Supplemental Table 2. Correlations of daytime napping with cognitive performance in Chinese elderly persons at each time point during 2011-2018.
Supplemental Table 3. Characteristics of naptime variation grouping among participants whose durations were 30-90 minutes at baseline included in GLMs
Supplemental Table 4. Supplemental Table 4. Characteristics of naptime variation grouping among participants whose napping =0, <30 minutes or >90 minutes at baseline included in GLMs
Supplemental Table 5. Associations between baseline daytime napping and longitudinal global cognition according to generalized estimating equations (GEE) between male and female.
Supplementary Figure 1. Plots of estimated smooth function of napping duration in Wave 1 with 95% confidence intervals for the GAM when the response variable was global cognition in Wave 4.

Supplemental Table 1. Definitions of variables used

Baseline characteristics	Wave	Definition
Time-invariant	Wave 1 (2011-2012)	
Age		Continuous
Gender		Male=1, Female=2
BMI		Continuous
High level of education		Low levels of education (without high school education or further study) =0, high levels of education (high school or more) =1
Smoking		Yes = 1, no = 2
Current alcohol use		Drinking at least once per week over the previous year = 1, no = 2
Hypertension		Yes = 1, no = 2
Dyslipidemia		Yes = 1, no = 2
Diabetes or high blood sugar		Yes = 1, no = 2
Cancer		Yes = 1, no = 2
Chronic lung diseases		Yes = 1, no = 2
Liver disease		Yes = 1, no = 2
Heart problems		Yes = 1, no = 2
Stroke		Yes = 1, no = 2
Kidney disease		Yes = 1, no = 2
Stomach or other digestive disease		Yes = 1, no = 2
Emotional, nervous, or psychiatric problems		Yes = 1, no = 2
Memory-related disease		Yes = 1, no = 2
Arthritis or rheumatism		Yes = 1, no = 2
Asthma		Yes = 1, no = 2
Vigorous physical activities		More than 10 minutes per week = 1, less than 10 minutes per week = 0
Moderate physical activities		More than 10 minutes per week = 1, less than 10 minutes per week = 1
Walking		More than 10 minutes per week = 1, less than 10 minutes per week = 2
Depression		without depression symptoms =0, defined as depression symptoms =1
Taking sleeping pills		Yes = 1, no = 0
Time-variant	Wave 1-4 (2011-2018)	
Cognitive performance score		Continuous variable ranging from 0 to 31
Napping		Continuous

Abbreviation: BMI, Body mass index

Supplemental Table 2. Correlations of daytime napping with cognitive performance in Chinese elderly persons at each time point during 2011-2018.

	NP2011	NP2013	NP2015	NP2018	Cog2011	Cog2013	Cog2015	Cog2018
NP2011	1							
NP2013	0.425[a]	1						
NP2015	0.460 [a]	0.486[a]	1					
NP2018	0.374[a]	0.384[a]	0.438[a]	1				
Cog2011	0.069[a]	0.053[a]	0.047[a]	0.044[a]	1			
Cog2013	0.069[a]	0.057[a]	0.047[a]	0.036[b]	0.622[a]	1		
Cog2015	0.072[a]	0.055[a]	0.039[b]	0.037[b]	0.631[a]	0.685[a]	1	
Cog2018	0.056[a]	0.049[a]	0.022	0.022	0.615[a]	0.664[a]	0.717[a]	1

Abbreviations: NP Napping, Cog Cognition.

[a] $P < 0.01$

[b] $P < 0.05$

Supplemental Table 3. Characteristics of naptime variation grouping among participants whose durations were 30-90 minutes at baseline included in GLMs

	Variations in nap duration				
	Decreased by > 0.5 h	Decreased by 0-0.5 h	No change	Increased by 0-0.5 h	Increased by > 0.5 h
Global cognition in Wave 2[a]					
Number of participants	299	155	294	124	160
Cognition mean (SD) score	11.78 (6.40)	12.37 (6.64)	13.67 (6.39)	12.69 (7.44)	12.74 (6.94)
Global cognition in Wave 3[b]					
Number of participants	275	146	313	131	167
Cognition mean (SD) score	11.08 (6.40)	11.43 (7.00)	12.37 (6.41)	11.92 (7.01)	10.92 (6.34)
Global cognition in Wave 4[c]					
Number of participants	287	134	304	121	186
Cognition mean (SD) score	8.32 (7.60)	11.43 (7.81)	10.15 (7.41)	11.06 (7.08)	7.63 (7.04)

[a] Change in napping presented the variations between napping in wave 2 and napping at baseline, being divided into 5 groups: decreased >0.5h/d, decrease 0-0.5h/d, no change, increased 0-0.5 h/d, and increased>0.5h/d, the GLMs showed the associations between this change in napping and global cognition scores in wave 2.

[b] Change in napping presented the variations between napping in wave 3 and napping at baseline, being divided into 5 groups: decreased >0.5h/d, decrease 0-0.5h/d, no change, increased 0-0.5 h/d, and increased>0.5h/d, the GLMs showed the associations between this change in napping and global cognition scores in wave 3.

[c] Change in napping presented the variations between napping in wave 4 and napping at baseline, being divided into 5 groups: decreased >0.5h/d, decrease 0-0.5h/d, no change, increased 0-0.5 h/d, and increased>0.5h/d, the GLMs showed the associations between this change in napping and global cognition scores in wave 4.

Supplemental Table 4. Characteristics of naptime variation grouping among participants whose napping =0, <30 minutes or >90 minutes at baseline included in GLMs

	Excessive[a]	No change[b]	Benefit 1[c]	Benefit 2[d]	Benefit 3[e]
No napping in Wave 1					
Number of participants	770	678	254	92	47
Cognition mean (SD) score	7.83 (7.02)	8.64 (6.92)	8.00 (7.00)	9.52 (7.80)	8.57 (7.40)
Short napping in Wave 1					
Number of participants	456	40	71	59	40
Cognition mean (SD) score	8.70 (7.22)	12.25 (7.19)	14.11 (8.08)	9.85 (7.68)	13.47 (8.61)
Long napping in Wave 1					
Number of participants	66	279	70	40	26
Cognition mean (SD) score	8.63 (7.31)	9.35 (7.02)	8.90 (7.42)	10.20 (7.41)	10.81 (8.21)

[a] Excessive, for participants with No napping in Wave 1, they were divided into Excessive group as long as they were in No or Long napping group once from Wave 2 to Wave 4; for participants with Short napping

at baseline, they were divided into Excessive group as long as they were in No or Long napping group once from Wave 2 to Wave 4; for participants with Long napping at baseline, they were divided into Excessive group as long as they were in No or Long napping group once from Wave 2 to Wave 4

[b] No change, for participants with No napping in Wave 1, they were divided into No change group if they maintained in No napping group from Wave 2 to Wave 4; for participants with Short napping at baseline, they were divided into No change group if they maintained in Short napping group once from Wave 2 to Wave 4; for participants with Long napping at baseline, they were divided into No change group if they maintained in Long napping group once from Wave 2 to Wave 4

[c] Benefit 1, for participants with No napping in Wave 1, they were divided into Benefit 1 group if they maintained No napping in two waves and had an Moderate napping in another wave; for participants with Short napping at baseline, they were divided into Benefit 1 group if they maintained Short napping in two waves and had an Moderate napping in another wave; for participants with Long napping at baseline, they were divided into Benefit 1 group if they maintained an Long napping in two waves and had an Moderate napping in another wave

[d] Benefit 2, for participants with No napping in Wave 1, they were divided into Benefit 2 group if they maintained in No napping group for one wave and in Moderate napping group for other two waves; for participants with Short napping at baseline, they were divided into Benefit 2 group if they maintained an Short napping in one wave and had two Moderate napping in other two waves; for participants with Long napping at baseline, they were divided into Benefit 2 group if they maintained an Long napping in one wave and had two Moderate napping in other two waves

[e] Benefit 3, for participants with No napping in Wave 1, they were divided into Benefit 3 group if they maintained in Moderate napping in three waves; for participants with Short napping at baseline, they were divided into Benefit 3 group if they Moderate napping in three waves; for participants with Long napping at baseline, they were divided into Benefit 3 group if they Moderate napping in three waves

Supplemental Table 5. Associations between baseline daytime napping and longitudinal global cognition according to generalized estimating equations (GEE) between male and female.

	Unstandardized β coefficient (95% confidence interval)			
	Model 1		Model 2	
	Male (1949)	Female (2075)	Male (1949)	Female (2075)
Categorical trend 1				
No napping	Ref.	Ref.	Ref.	Ref.
Short napping	0.351 (-0.316, 1.019)	**2.011 (1.346, 2.675)***	0.275 (-0.346, 0.896)	**1.326 (0.661, 1.990)***
Moderate napping	0.768 (0.223, 1.312)**	**0.651 (0.071, 1.231)***	0.436 (-0.082, 0.953)	-0.023 (-0.887, 0.841)
Long napping	-0.072 (-0.724, 0.579)	0.601 (-0.209, 1.411)	-0.089 (-0.695, 0.517)	0.270 (-0.868, 1.409)
Categorical trend 2				
No napping	Ref.	Ref.	Ref.	Ref.
Napping	0.455 (0.001, 0.909)	**1.130 (0.669, 1.591)***	0.267 (-0.160, 0.694)	0.459 (-0.025, 0.943)

***P<0.001; **P<0.01; *P<0.05

Model 1: adjusted for age.

Model 2: adjusted for Model 1+ educational levels, physical activities, BMI, depression, use of tranquilizers, smoking, alcohol consumption, night sleep duration at baseline, medical history including hypertension, dyslipidemia, heart disease and other 11 chronic diseases.

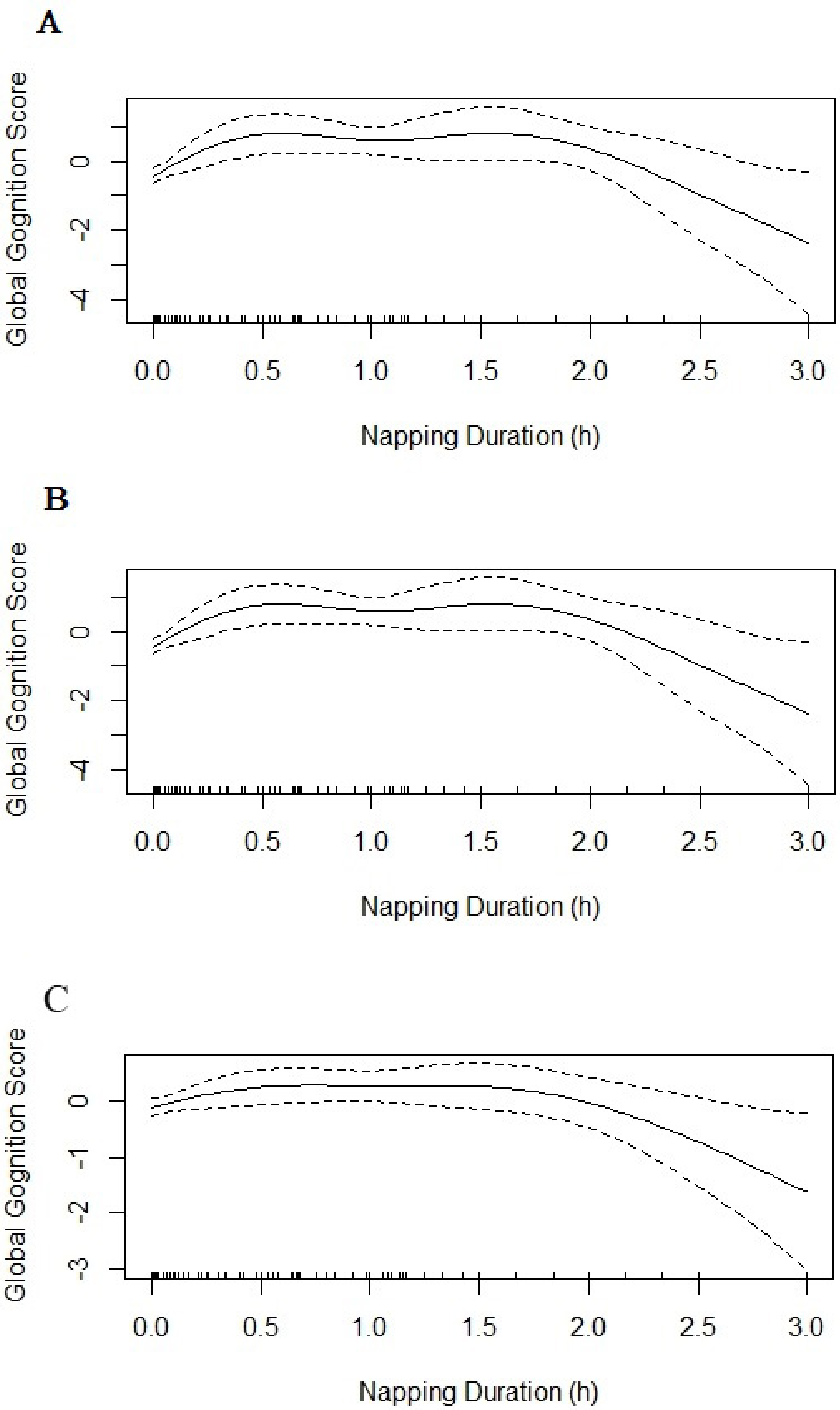

Supplementary Figure 1. Plots of estimated smooth function of napping duration in Wave 1 with 95% confidence intervals for the GAM when the response variable was global cognition in Wave 4. The range between the dashed lines indicates the 95% confidence interval of the estimated smooth function, and the solid lines indicate the estimated smooth function of napping duration in Wave 1 for the GAM. (A) Model 0 showed a univariate smooth function of napping duration (EDF =

3.754, $P < 0.001$). (B) Model 1 presented a multivariable smooth function of napping duration, adjusted for age and sex (EDF = 4,217, $P < 0.001$). (X) Model 2 presented a multivariable smooth function of napping duration, adjusted for age, sex educational levels, physical activities, BMI, depression, use of tranquilizers, smoking, alcohol consumption, night sleep duration at baseline, medical history including hypertension, dyslipidemia, heart disease and other 11 chronic diseases (EDF = 2.843, $P = 0.046$).
